# The Diagnostic Relevance and Interfaces Covered by Mach Band Effect in Dentistry: An Analysis of the Literature

**DOI:** 10.3390/healthcare10040632

**Published:** 2022-03-28

**Authors:** Andy Wai Kan Yeung

**Affiliations:** Oral and Maxillofacial Radiology, Applied Oral Sciences and Community Dental Care, Faculty of Dentistry, The University of Hong Kong, Hong Kong SAR, China; ndyeung@hku.hk

**Keywords:** mach band effect, mach effect, optical illusion, caries, dental restoration, dental implant

## Abstract

This work surveyed how the Mach band effect was mentioned in the dental literature and provided a qualitative assessment of diagnostic relevance and interfaces covered. PubMed, Scopus, and Google Scholar were queried in mid-Jan 2022. The search string was (“mach band effect” OR “mach effect”) AND (dental OR oral OR tooth OR teeth OR maxillofacial OR orofacial). All publications returned by the searches were screened. Exclusion criteria included irrelevance (e.g., dealing with “Mach effect” that was non-radiographic or non-dental) and not written in English. Reference lists of returned publications were manually searched to identify potentially missed papers. For each included publication, the following parameters were recorded: any presentation of radiographic images showing a Mach band effect, direct investigation of the effect, relevance to which structural interfaces, diagnostic relevance, and in which parts of the publication Mach band effect was mentioned. Seventy-seven publications were included and analyzed. The majority of the publications mentioned the Mach band effect in the Discussion section about its diagnostic relevance to caries detection at the enamel-dentinal junction and the interface between restorative material and tooth structure. Eight of them presented radiographic images showing a Mach band effect. Three of them investigated the Mach band effect. Dental publications seldom covered the Mach band effect. When they covered it, most of them only mentioned it in the Discussion section without actually investigating it.

## 1. Introduction

The Mach band effect was first described by Austrian physicist Ernst Mach in 1865 [1]. It is an optical illusion caused by an inherent edge enhancement by the retina, resulting in a darker edge of a dark object adjacent to a light object, and vice versa. The apparent change can be proved as an optical illusion by photodensitometric tracing, as the latter will demonstrate a consistent reading without alterations at the affected area [2]. The apparent alteration in the perceived color has important implications for medical radiology. For example, the Mach band effect could produce a dark halo surrounding a dense breast lesion in mammography [3], present a pseudofracture of the odontoid process in the cervical spine [4], and mimic pneumomediastinum [5]. The relevance of the Mach band effect to dental radiology was pointed out by Berry Jr. [6], who noted that Mach bands and cervical burnout are two common phenomena producing radiolucency that resemble carious lesions. In the distant past, it was even suggested that the lamina dura visible on dental radiographs was caused by the Mach band effect [7], only to be refuted later by densitometric measurements [8].

The Mach band effect seems to be common and could occur in many clinical situations [6,9]. In these circumstances, radiologic expertise would be necessary, and such superior perception should be defined by improved visual search patterns and diagnostic accuracy for a better efficacy in deciding whether a feature should be attended or ignored and, eventually, abnormality detection [10,11,12]. However, it was largely unknown how the dental academic literature has considered or investigated the Mach band effect. Therefore, the main purpose of this literature survey was to evaluate how many publications have mentioned and investigated it respectively. More importantly, this survey evaluated the structural interfaces covered, diagnostic relevance, and location in the publications where the Mach band effect was mentioned.

## 2. Materials and Methods

**Figure 1:**
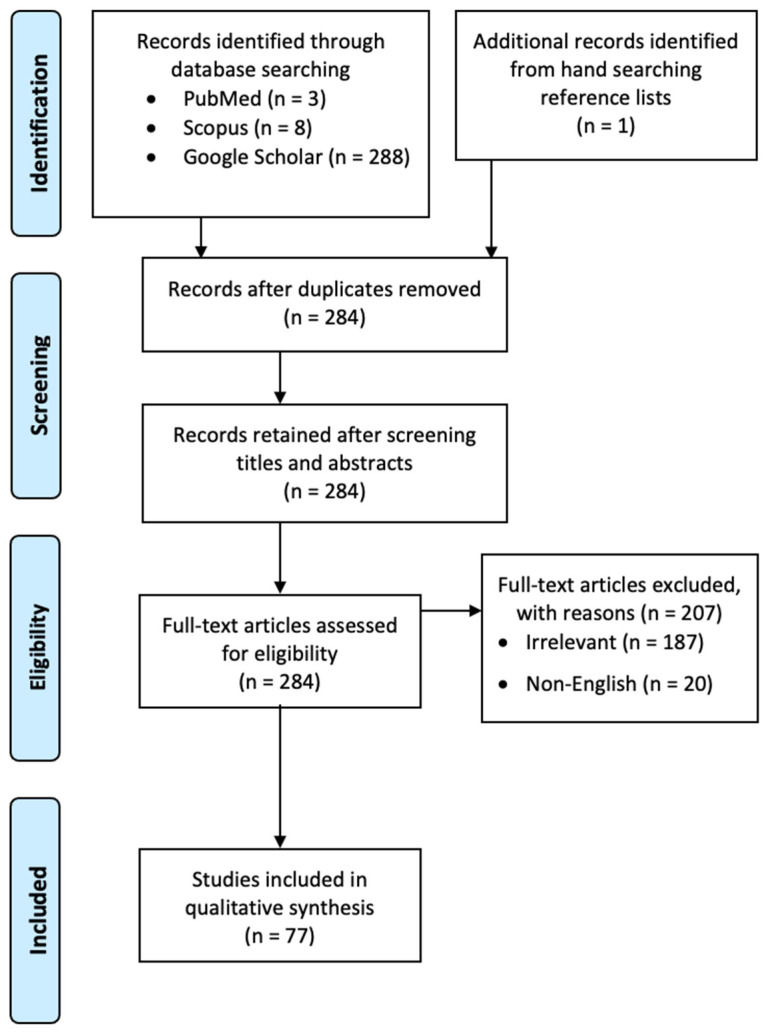
A PRISMA flow chart showing the screening process of the literature search.

On 20 January 2022, PubMed, Scopus, and Google Scholar were queried. The search string was: (“mach band effect” OR “mach effect”) AND (dental OR oral OR tooth OR teeth OR maxillofacial OR orofacial). To enable batch export, Google Scholar was queried with a software called Publish or Perish (https://harzing.com/resources/publish-or-perish (accessed on 20 January 2022)). All three literature databases searched all fields of the publications (i.e., not limited to title, abstract and keywords), but Google Scholar yielded far more publications due to its broadest literature coverage as well as its ability to gain access to the full text of the publications (Figure 1). All publications returned by the searches were initially included. The content of each one was manually checked, and a publication was excluded if it was irrelevant (e.g., dealing with “Mach effect” that was non-radiographic or non-dental) or written in non-English. To maximize coverage, this literature survey did not exclude books and dissertations resulting from the search.

The entire literature screening process is illustrated in Figure 1. Finally, 77 publications were included and analyzed. Ethical approval was not applicable to this literature survey.

## 3. Results

**Figure 2:**
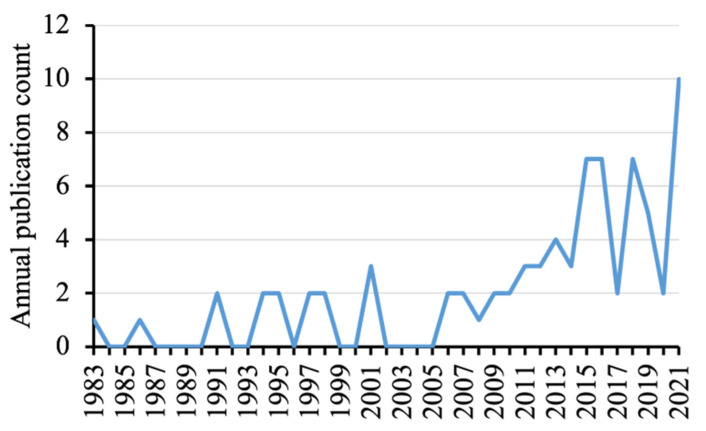
Annual publication count that mentioned Mach band effect.

The 77 publications that mentioned the Mach band effect were mainly experimental (original) articles (*n* = 53, 68.8%), whereas the others were review/opinion papers (*n* = 13, 16.9%), dissertations (*n* = 5, 6.5%), and academic books (*n* = 6, 7.8%). The first publication could be traced back to 1983 by Berry Jr. [6]. There seemed to be more publications that mentioned the Mach band effect towards the late 2010s (Figure 2). The full details of the 77 publications are provided in Supplementary Table S1.

Eight publications (five original articles, two review papers, one book) presented dental radiographic images that illustrated the Mach band effect [9,13,14,15,16,17,18,19]. Three original articles investigated the Mach band effect as follows. In 2001, Nielsen [9] found that students were more likely to misinterpret a Mach band present at the junction of the alveolar crestal bone and the root of a maxillary central incisor as a horizontal root fracture line compared to dentists (17.6% vs. 9.5%); whereas many more students and dentists misinterpreted when the radiograph was presented with a scenario of recent trauma (62.5% vs. 21.1%). In 2011, Qaramaleki and Hassanpour [17] evaluated the use of the adaptive gamma correction method to process panoramic images to reduce the Mach band effect for better caries diagnosis. In 2017, Movahhedian et al. [20] revealed that nearly 80% of dental students and dentists interpreted non-carious triangular-shaped radiolucency (cf. [21]) as a sound surface or a Mach band.

In terms of structural interface, the most frequently mentioned locations where the Mach band effect could occur were (1) between very radiopaque restorative material and tooth structure, and (2) at the enamel-dentinal junction (Table 1). The Mach band effect was also often mentioned to happen between dental implant and its surrounding bone (peri-implant tissue). Meanwhile, the Mach band effect at the interface between root filling and root structure was rarely mentioned. In terms of diagnostic relevance, the most frequently mentioned item was resemblance to a carious lesion. Others were much less mentioned, such as resemblance to a marginal defect (of a restoration), void in root filling, root fracture, and capsule around a lesion.

Another five publications (three original articles and two dissertations) merely mentioned the definition or the effect brought by the Mach band without relating it to structural interface or diagnostic relevance.

Finally, the 53 original articles usually mentioned the Mach band effect in their Discussion section (Table 2). Few of them mentioned it in the Methods, Results, or Conclusion.

## 4. Discussion

This literature survey identified 77 academic publications in dentistry that mentioned the Mach band effect, ranging from original articles, review papers, books and dissertations. Most of them mentioned that the Mach band effect might create apparent radiolucency at the enamel-dentinal junction or adjacent to a radiopaque restorative material, which might resemble a carious lesion. Few of them presented dental radiographic images to illustrate the appearance of Mach bands, and even fewer of them really investigated this matter.

The results seemed to suggest that the Mach band effect was quite well-known to the dental community, and it became a common explanation in the Discussion section for false positive observations in caries detection:

- “*The radiographs were interpreted with caution, bearing in mind the possibility of false-positive diagnoses that might arise from the Mach-band effect…*” [22]
- “*A tendency to make false-positive scores is common, and this could be due to the Mach-band effect…*” [23]
- “*A high radio-opacity near a less radioopaque area can cause the Mach Band effect… This effect might be misinterpreted as caries…*” [24]

The finding that 73.6% of the articles that mentioned Mach band effect in the Discussion section was not unexpected. In fact, a quick search in the Scite database [25] found that the pioneer work by Berry Jr. that described a Mach band and cervical burnout in dental radiology was largely cited in the Discussion section of citing papers. For 10 citation statements extracted from the Introduction, Methods, Results, and Discussion of papers indexed in the Scite database, 80% of them cited Berry Jr. in their Discussion and all of them mentioned the reference without supporting or contrasting semantic contexts. In other words, those citing papers did not “test” [26] the theory or findings regarding the Mach band effect from Berry Jr. [6].

Since the Mach band effect is an optical illusion, it is possible to “mask” or circumvent it. As Berry Jr. [1] suggested, if a radiolucency was present in the dentine underneath the enamel-dentinal junction, masking the enamel with an opaque card or piece of paper would negate the lateral inhibitory effect and hence eliminate the Mach band. After removing the mask, the Mach band would reappear. If the radiolucency was always present regardless of the mask, then the radiolucency was not a Mach band. The critical point is to mask the interface between the radiopaque and less radiopaque structure. However, such a method was not adopted by studies that mentioned the Mach band effect in the Methods section. Instead, verbal caution or a customized criterion was used:

- “*Observers had to score voids within the root filling and not around it. The artefactual burnout phenomena (Mach bands) seen on CBCT images around root fillings were explained and the observers were told not to score them as a void…*” [14]
- “*To avoid false positive diagnoses caused by the Mach band effect, only obvious radiolucencies in the dentin were scored as dentin caries. This applied in particular to small radiolucencies adjacent to the enamel-dentin border of occlusal surfaces; surfaces with such radiolucencies were scored as sound…*” [27]
- “*Since the contrast between enamel and dentin may result in the Mach-band effect, the suspected area was carefully scrutinized and compared to the DEJs [enamel-dentinal junction] in the other teeth on the patient’s bitewings…*” [16]

Therefore, one clinically relevant aim of this work was to remind clinicians to be aware of the Mach band effect during radiographic image interpretation, which was not limited to resemblance to caries or marginal defect of a restoration, but also to a variety of scenarios listed above. Moreover, as Buckle et al. [28] explained, visual illusion could occur in three levels: image formation, sensation, and perception. At the image formation level, modality specific artifacts such as a parallax effect might occur. Here, a parallax effect meant an apparent exaggerated relative position (distance) between two objects that could lead to misinterpretation and misdiagnosis. At the sensation level, the Mach band effect and background effect might occur. The latter meant that the optical density or gray level of an object could be modulated by its background. In other words, two identical objects might look differently, depending on whether the background was light or dark. Finally at the perception level, ambiguous figures, fictional illusions, and other perceptual illusions might exist. Notable ambiguous figures in the popular culture included “mother or wife” and “faces or vase”. Fictional illusions meant the apparent presence of an object that was actually absent. Meanwhile, one of the other perceptual illusions was distortion (e.g., size), well-illustrated by the Ebbinghaus illusion or Titchener circles [29]. It meant that two equal-sized radiographic lesions might look very different in size unless the observer actually placed a ruler to measure their dimensions. All these examples showed that our visual sensation and perception might not be reliable at all times during radiologic interpretation. Clinicians, not only radiologists, should be aware of all these potential visual illusions that can appear on radiographic images during image interpretation and diagnosis. Meanwhile, the potential dental radiologic applications of artificial intelligence such as for caries detection may circumvent the issue with Mach bands [30], but clinicians still need to be aware of its potential presence so that they will not wrongly overrule the software decision.

This literature survey only considered English-language publications. Readers should be aware that the findings might not be readily applicable to the non-English literature.

## 5. Conclusions

This literature survey identified fewer than a hundred dental publications that mentioned the Mach band effect. Very few of them presented radiographic images that illustrated the Mach band effect or actually investigated the Mach band effect. Instead, the majority of the publications mentioned the Mach band effect in the Discussion section about its diagnostic relevance to caries detection at the enamel-dentinal junction and the interface between restorative material and tooth structure. Beside restorative/cariology relevance, the Mach band effect was also mentioned with relevance to implant dentistry and endodontology. However, no study was found to adopt a masking technique to eliminate the Mach band effect as the observers interpreted the images, which has been suggested by earlier works [6,28]. Dentists are increasingly aware of the Mach band effect. There is evidence of diagnostic relevance, but few studies have tried to circumvent the effect or otherwise improve diagnostic accuracy in situations where the effect occurs.

## Figures and Tables

**Table 1:** Frequency counts of structural interface and diagnostic relevance mentioned by the analyzed publications that mentioned Mach band effect.

	No. of Publications	% (of 77)
(A) Structural interface		
Implant/bone	10	13.0
Restoration/tooth	28	36.4
Root filling/root canal	3	3.9
Enamel-dentinal junction	21	27.3
(B) Diagnostic relevance		
Relevance to edge detection	1	1.3
Mimics caries	47	61.0
Mimics marginal defect	5	6.5
Mimics void in root filling	3	3.9
Mimics triangular-shaped radiolucency	2	2.6
Mimics root fracture	2	2.6
Mimics fracture at mandibular angle	1	1.3
Mimics a capsule around a lesion	2	2.6

**Table 2:** Frequency counts of article sections where the original articles mentioned Mach band effect.

	No. of Publications	% (of 53)
Introduction	13	24.5
Methods	7	13.2
Results	2	3.8
Discussion	39	73.6
Conclusion	1	1.9

## Data Availability

All data is available in the manuscript.

## Supplementary Materials

The following supporting information can be downloaded at: https://www.mdpi.com/article/10.3390/healthcare10040632/s1, Table S1: Full list of the 77 analyzed publications.
